# The Build-Up and Transfer of Sensorimotor Temporal Recalibration Measured via a Synchronization Task

**DOI:** 10.3389/fpsyg.2012.00246

**Published:** 2012-07-12

**Authors:** Yoshimori Sugano, Mirjam Keetels, Jean Vroomen

**Affiliations:** ^1^Faculty of Management, Kyushu Sangyo UniversityFukuoka, Japan; ^2^Department of Psychology, Tilburg UniversityTilburg, Netherlands

**Keywords:** adaptation, transfer, temporal recalibration, tapping, sensorimotor, crossmodal, delayed visual feedback, delayed auditory feedback

## Abstract

The timing relation between a motor action and the sensory consequences of that action can be adapted by exposing participants to artificially delayed feedback (temporal recalibration). Here, we demonstrate that a sensorimotor synchronization task (i.e., tapping the index finger in synchrony with a pacing signal) can be used as a measure of temporal recalibration. Participants were first exposed to a constant delay (~150 ms) between a voluntary action (a finger tap) and an external feedback stimulus of that action (a visual flash or auditory tone). A subjective “no-delay” condition (~50 ms) served as baseline. After a short exposure phase to delayed feedback participants performed the tapping task in which they tapped their finger in synchrony with a flash or tone. Temporal recalibration manifested itself in that taps were given ~20 ms earlier after exposure to 150 ms delays than in the case of 50 ms delays. This effect quickly built up (within 60 taps) and was bigger for auditory than visual adapters. In Experiment 2, we tested whether temporal recalibration would transfer across modalities by switching the modality of the adapter and pacing signal. Temporal recalibration transferred from visual adapter to auditory test, but not from auditory adapter to visual test. This asymmetric transfer suggests that sensory-specific effects are at play.

## Introduction

Timing of an action is crucial in daily activities like stepping on an escalator, catching a ball, playing a musical instrument, dancing, or playing video games. In all these examples, we have learned to correctly time a voluntary action through a lifetime’s experience. The environment and individuals, though, are also changing and it is therefore of benefit to adapt to new sensorimotor timing relationships in response to these changes. The way the brain adjusts to these new timing relations, though, is still unclear.

Experimental psychology has a long history in investigating the plasticity of sensorimotor coordination. The most famous one is prism adaptation (Stratton, 1897) in which there is a re-arrangement of spatial visuo-motor relations. Much less known, though, is that the timing between an action and its sensory consequence is also flexible, and that adaptation to temporal delays can ultimately lead to an illusory reversal of the cause-effect order. In a study by Cunningham et al. (2001), this was demonstrated by having participants adapt to delayed visual feedback after a voluntary movement. When the visual delay was removed, participants reported that the visual cursor appeared to move before the motor act. Stetson et al. (2006) demonstrated this more formally by asking observers to judge the temporal order of a tap and a flash after exposure to 35 and 135 ms delays between voluntary taps and subsequently delivered light flashes. Participants adapted to the longer delays and judged unexpectedly short delays to occur before the actual tap (see also Heron et al., 2009, for audio-motor and tactile-motor recalibration). Temporal recalibration is not only restricted to artificial stimuli like flashes and beeps, but has also been found with natural stimuli as in delayed auditory feedback of speech (Yamamoto and Kawabata, 2011) and in delayed visual feedback of natural hand movements (Keetels and Vroomen, 2012). It is also of note that temporal recalibration has parallels with purely *sensory* effects observed following adaptation to audio-visual, audio-tactile, and visuo-tactile asynchrony (Fujisaki et al., 2004; Vroomen et al., 2004; Harrar and Harris, 2005, 2008; Navarra et al., 2007; Hanson et al., 2008; Keetels and Vroomen, 2008; Takahashi et al., 2008).

The cognitive and neural mechanisms of temporal recalibration, though, have not been fully specified. One hypothesis is that a single supramodal mechanism, which usually refers to a “central clock” model, is responsible for the recalibration of perceived time across sensory pairings. The central clock refers to a dedicated single, centralized internal time keeper mechanism in which pulses are generated by a pacemaker and are counted by a counter (Creelman, 1962; Treisman, 1963). This idea is in line with data showing equal amounts of temporal recalibration across all auditory, visual, and tactile sensory pairings (Hanson et al., 2008). Support for this concept also comes from studies showing that motor-sensory temporal recalibration readily transfers between sensory modalities (Heron et al., 2009; Sugano et al., 2010), and transfers from learned to novel tasks (Fujisaki et al., 2004; Pesavento and Schlag, 2006). The latter findings, though, are not unequivocal evidence in favor of a central supramodal clock since it may also be the case that participants have changed a *unimodal* criterion (criterion for solely one sensory modality) in one of the involved modalities, for example about when they have initiated an action or when they have perceived a sensory event.

There is also other evidence that is difficult to reconcile with a centralized clock model and that rather points toward early, peripheral timing mechanisms that are selective for modality and low-level stimulus features. For example, some reported a complete absence of recalibration outside the audio-visual domain (Navarra et al., 2007; Harrar and Harris, 2008), while others reported relatively lower levels of visuo-tactile recalibration (Takahashi et al., 2008). The magnitude of audio-motor adaptation has also been found to be greater than visual-motor and tactile-motor adaptation, and there are also costs involved when the modality of the sensory event changes between the adaption and test phase (Heron et al., 2009). The notion of a central clock is also difficult to reconcile with the finding that observers can have *multiple* concurrent estimates of audio-visual synchrony for different pairings, and that temporal recalibration can occur in positive and negative directions concurrently (Roseboom and Arnold, 2011; Heron et al., 2012). There may also be a role for attention as attending to the temporal structure of asynchronous auditory and visual adapting stimuli during adaptation can increase temporal recalibration induced by these stimuli, possibly since it increases the saliency of the temporal pattern (Heron et al., 2010).

**Figure 1:**
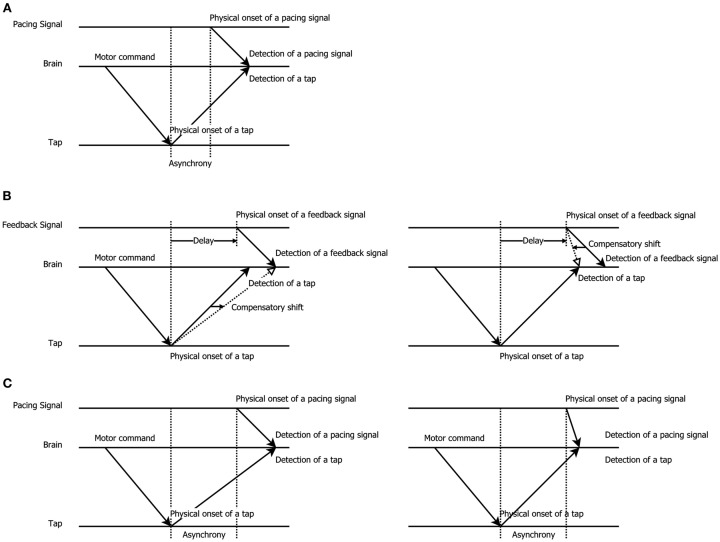
**Schematic illustration of the shift in tapping asynchrony after exposure to delayed feedback, modified from Aschersleben and Prinz (1997)**. **(A)** *Synchronous tapping before adaptation:* the latency difference between detection of a pacing signal (sound or flash) and tap gives rise to a tap asynchrony (tap-before-pacing-signal; Paillard–Fraisse hypothesis, Paillard, 1949; Fraisse, 1980; Aschersleben and Prinz, 1997). **(B)** *Exposure to delayed feedback*: to re-align the delayed external feedback after a voluntary tap participants may shift the representation of when the pad was touched (left panel) or when the pacing signal came (right panel), thus causing adaptation. **(C)** *Synchronous tapping after adaptation*: taps are given earlier due to the lingering effect of adaptation to delay that either slowed down the detection of a tap (left panel) or sped-up the detection of the pacing signal (right panel).

The variability in these results raises the question how temporal recalibration is best characterized and whether it should be described as mandatory or cognitive in nature. This also forces one to more closely examine the way temporal recalibration is assessed. The temporal order judgment (TOJ) task, in which participants decide which stimulus appeared first/last, and the simultaneity judgment (SJ) task, in which participants decide whether stimuli were simultaneous or not, are by far the most popular tests of temporal recalibration. Though, several authors have argued that these explicit tasks about order or synchrony are not always optimal. The TOJ task may be susceptible to response biases since participants can deliberately shift their criterion about which stimulus came first and they may also try to equate their responses over the available response categories (Schneider and Bavelier, 2003; van Eijk et al., 2008). The SJ task is also not free from biases since participants may deliberately change their criterion for synchrony (see Vroomen and Keetels, 2010 for review). Here, we therefore wanted to examine an *indirect* test of temporal recalibration (a test not directly asking participants about a temporal relationship), namely a sensorimotor synchronization task in which observers tap their finger in synchrony with an external pacing signal (*implicit timing*, Coull and Nobre, 2008; Coull et al., 2011). The innovative aspect of using this well-known tapping task is that before observers commenced tapping, they first adapted to delayed feedback of this action. We predicted that temporal recalibration would manifest itself as a compensatory shift in the natural negative asynchrony (tap-before-pacing-signal) between the tap and pacing signal: observers adapted to delayed feedback thus were expected to tap *earlier* to compensate the previously experienced delay (see Figure 1 for a graphic explanation).

It would be an important methodological improvement if it can indeed be demonstrated that temporal recalibration can be measured via tapping asynchrony. The advantage of tapping is that it is a relatively easy and natural task that is free from explicit response biases since participants are not required to make explicit judgments about temporal order as in the SJ or the TOJ task. The other merit of tapping is that it allows one to determine the time course of the effect (e.g., its build-up and dissipation) more precisely since it requires only a few taps to get a relatively stable measure of tap-stimulus asynchrony, whereas the SJ and the TOJ tasks require many trials to obtain a reliable psychometric function. Finally, there is an extensive literature on tapping (for reviews, see Aschersleben, 1999, 2002; Repp, 2005), though not in the context of temporal recalibration.

Our goal was to first determine whether tapping can indeed serve as a sensible measure of temporal recalibration. To do so, observers adapted, as in previous studies on motor-sensory recalibration (Stetson et al., 2006; Heron et al., 2009; Sugano et al., 2010; Stekelenburg et al., 2011; Keetels and Vroomen, 2012), to a fixed delay (either 50 or 150 ms) between the completion of a voluntary motor action (a finger tap) and either a visual (a white flash on a computer screen) or auditory (a tone “pip”) external feedback signal of that action. The critical idea is that this delayed feedback shifts the central representation of the motor signal (when did I move the finger) or kinesthetic/tactile information (when did I touch the pad) in time forward toward the delayed signal (Aschersleben and Prinz, 1997, Experiment 2). After a short exposure phase to the delayed feedback, participants then tapped their finger in the test phase in synchrony with a pacing signal. We predicted that temporal recalibration would manifest itself as a compensatory shift in the asynchrony between the tap and the pacing signal: observers adapted to long delays thus should tap *earlier* after adaptation than prior to adaptation. The difference in the average tap asynchrony before and after adaptation served as our primary measure of temporal recalibration. The task also allows us to address whether – according to the notion of a supramodal central clock – the overall magnitude and build-up of motor-auditory and motor-visual temporal recalibration is comparable (Experiment 1), and whether there is equal transfer from visual adapter to auditory test and vice versa (Experiment 2). Alternatively, if there are modality-specific differences in magnitude or build-up, or if there are costs involved in the transfer between modalities, it is likely that modality-specific mechanisms are at play.

## Experiment 1

### Material and methods

#### Participants

Thirty-four participants from Kyushu Sangyo University and 22 participants from Tilburg University (18 female, mean age 20.0, six left-handed, all using a computer mouse by their right hand) participated in the experiment. Approximately half of them (29 participants) received visual adaptor combined with visual test, the remaining participants received auditory adaptor combined with auditory test. All had normal hearing and normal or corrected-to-normal vision. Informed consent was obtained from each participant. The experiment was approved by the Local Ethics Committee of Kyushu Sangyo University and Tilburg University, and followed the declaration of Helsinki.

#### Stimuli and apparatus

Participants sat at a desk in a dimly lit and soundproof booth looking at a CRT display at approximately 65 cm viewing distance. The visual stimulus consisted of a 1-cm white square (9 cd/m^2^) flashed for 30 ms on a black background (0 cd/m^2^). The auditory stimulus consisted of a 2,000-Hz pure tone pip (30 ms duration, 2 ms rise/fall slope) presented via headphones at 74 dB(A). For catch trials (see Procedure), a 1-cm red square (3 cd/m^2^, 30 ms) and a 2,250-Hz pure tone pip [30 ms with 2 ms slope, 74 dB(A)] were used. White noise was continuously presented via headphones at 74 dB(A) to mask the sound of the taps. A special gaming mouse (Sanwa Supply MA-LSPRO and Logitech G500) was used for detecting the precise timing of the finger taps. The temporal resolution of the device was about 2 ms as verified by dedicated software (“Mouse Rate Checker” by Oliver Tscherwitschke). The timing of stimulus presentation was verified on a multiple trace oscilloscope.

#### Design

The modality of the test and adaptor (auditory or visual) was a between-subjects factor, while test type (pre- or post-test) and exposure delay (50 or 150 ms) were within-subjects factors, thus yielding eight different conditions. Each condition consisted of 30 trials. The two exposure delays were split across two consecutive days and counterbalanced for order across participants. The whole experiment lasted ~60 min including instruction, practice sessions, and experimental sessions.

#### Procedure

In the pre-test, participants pressed their index finger on the mouse in synchrony with a pacing signal (a flash or a tone). The pacing signal was delivered 10 times per trial at a constant inter-stimulus interval (ISI) of 750 ms. Participants skipped the first three signals to get into the rhythm, and then synchronized their mouse-clicks with the following seven signals. There were 30 trials in total.

After completion of the pre-test, the adaptation/post-test phase began. Each trial started with a short adaptation phase immediately followed by a post-test. In the adaptation phase, participants voluntarily pressed the mouse 10 times trying to keep the inter-tap interval at approximately 750 ms. After each tap, a feedback stimulus (a flash or a tone) was delivered at a constant lag of either 50 or 150 ms. These values were chosen since the tap-flash and tap-tone pairings were still perceived as a single event, and were expected to elicit quantifiable adaptive shifts (Sugano et al., 2010; Stekelenburg et al., 2011). To ensure that participants attended the feedback stimulus, they had to detect the occasional occurrence of a deviant stimulus (a red square or a high tone; subsequently referred to as catch trial). Participants were questioned at the end of the adaptation phase whether the deviant stimulus had been presented or not. In the post-test that immediately followed the adaptation phase, participants then performed the tapping task which was identical to the pre-test. Each adaptation/post-test phase contained 30 trials. A short practice session before the pre-test and another before the adaptation phase preceded the experiment.

### Results

Trials of the practice sessions were excluded from further analysis. Performance on the catch trials in the adaptation phase was almost flawless (99.4% correct) indicating that participants were indeed looking at the light or listening to the sound during adaptation. Missing responses were only 0.3% in total. Tap asynchronies (i.e., time difference between a tap and a pacing signal) out of the range from −250 to +100 ms were eliminated from further analyses (0.6% in total). The rest of the tap asynchronies (seven measurements per trial) were averaged over the 30 trials for each experimental condition.

#### Average tap – stimulus asynchronies

The group-averaged tap asynchronies of Experiment 1 are presented in Table 1 (upper panel). All of them were negative, which reflects the well-known anticipation tendency in sensorimotor synchronization (see, e.g., Aschersleben, 2002). The anticipation tendency was significantly greater with auditory than with visual pacing signals. Exposure to feedback without any noticeable delay (50 ms) did not change the anticipation tendency from pre- to post-test. Most importantly, the negative asynchrony became – as predicted – more negative after exposure to delayed auditory and visual feedback. The difference between pre- and post-test is referred to as the temporal recalibration effect (TRE).

**Table 1:** **Mean tap asynchronies**.

Experiment	Modality		Lag (ms)	Mean tap-stimulus asynchrony (ms)		Temporal recalibration effect (post – pre)
	Adaptor	Test		Pre	Post	
Experiment 1	Visual	Visual	50	−56.0 (5.1)	−53.1 (5.1)	2.9 (2.9)
			150	−54.8 (5.3)	−67.1 (5.5)	−12.3* (3.9)
	Auditory	Auditory	50	−84.0 (6.2)	−81.7 (5.3)	2.4 (4.2)
			150	−79.8 (6.3)	−104.2 (6.0)	−24.4** (4.5)
Experiment 2	Auditory	Visual	50	−50.7 (9.0)	−59.3 (9.6)	−8.6 (6.3)
			150	−45.8 (8.3)	−50.2 (8.3)	−4.5 (5.4)
	Visual	Auditory	50	−66.4 (7.0)	−74.8 (8.1)	−8.4 (4.5)
			150	−72.3 (8.2)	−103.4 (9.5)	−31.0** (4.8)

*Standard errors of means (SEMs) are shown in parentheses*.

*p < 0.01. **p < 0.001. (i.e., negative numbers indicate tap before auditory / visual stimulus).

To test these observation, a mixed-model ANOVA was conducted on the individual asynchronies with the modality of the test (visual vs. auditory) as a between-subjects factor, and with the test type (pre- vs. post-test) and the exposure delay (50 vs. 150 ms) as within-subjects factors. There was a main effect of the modality, *F*(1, 54) = 17.9, *p* < 0.001, since the anticipation tendency was more negative with auditory than with visual test (−87.4 vs. −57.7 ms, respectively). There were also main effects of the test type, *F*(1, 54) = 12.4, *p* < 0.001, the exposure delay, *F*(1, 54) = 9.8, *p* < 0.01, and an interaction between the test type × the exposure delay, *F*(1, 54) = 39.3, *p* < 0.001. No other effects were significant (*p* > 0.05). To analyze the test type × the exposure delay interaction, we subtracted the anticipation tendency in the post-test from the pre-test (the TRE), and ran separate *t*-test (one-sided as there was a clear prediction) on them (with Bonferroni corrected alpha set to 0.0125). As predicted, tapping asynchronies became more negative after exposure to delayed visual and auditory feedback [*t*(28) = 3.2, *p* < 0.01, and *t*(26) = 5.5, *p* < 0.001, respectively], however, there was no difference after exposure to non-delayed visual and auditory feedback [*t*(28) = 1.0, *p* = 0.84, and *t*(26) = 0.6, *p* = 0.71, respectively]. To test directly if the tap asynchrony under the delayed feedback became more negative compared to the non-delayed feedback, we ran paired *t*-tests (one-sided) on the tap asynchronies between delayed and non-delayed feedback for the visual and the auditory test (with Bonferroni corrected alpha set to 0.025). For both modalities, the tap asynchrony after delayed feedback was more negative than the non-delayed one, *t*(28) = 3.5, *p* < 0.001 for the visual feedback, *t*(26) = 5.4, *p* < 0.001 for the auditory feedback. The TREs of visual and auditory delayed adapters were also different from each other as auditory adapters induced a greater TRE than visual adapters [*t*(54) = 2.0, *p* < 0.05].

An additional mixed-model ANOVA was conducted on the TREs to check for a possible carryover effect between the two exposure delays, with the modality of the test and the execution order of the delay (lag-50 ms on the first day and lag-150 ms on the second day, or vice versa) as between-subjects factors, and with the exposure delay as a within-subjects factor. The ANOVA indicated that none of the effects regarding the execution order of the delay were significant (all *p*s > 0.05) confirming that there was no carryover effect between the two exposure delays.

#### Build-up and dissipation

**Figure 2:**
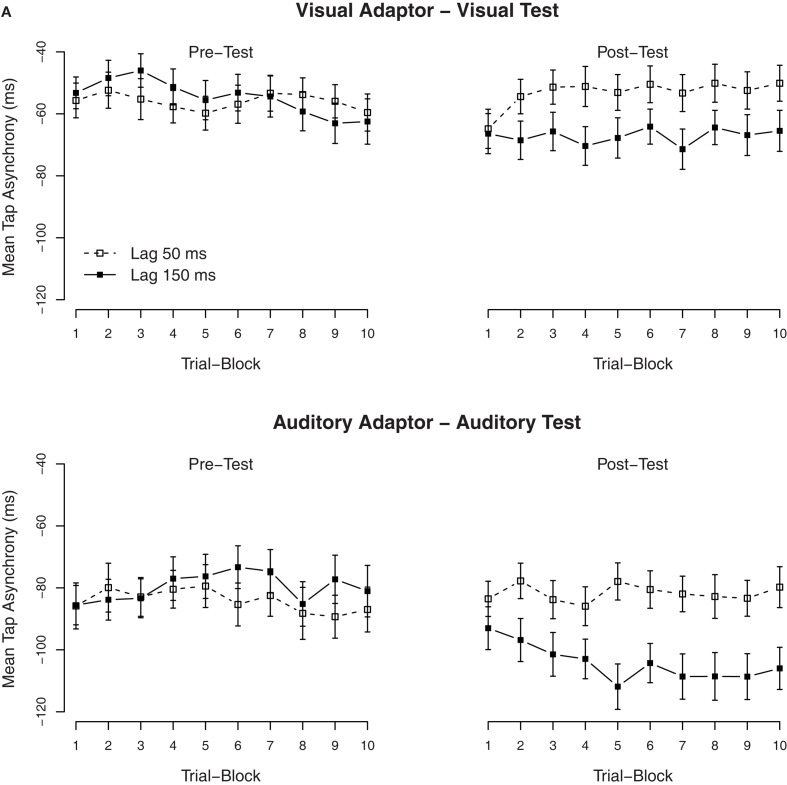

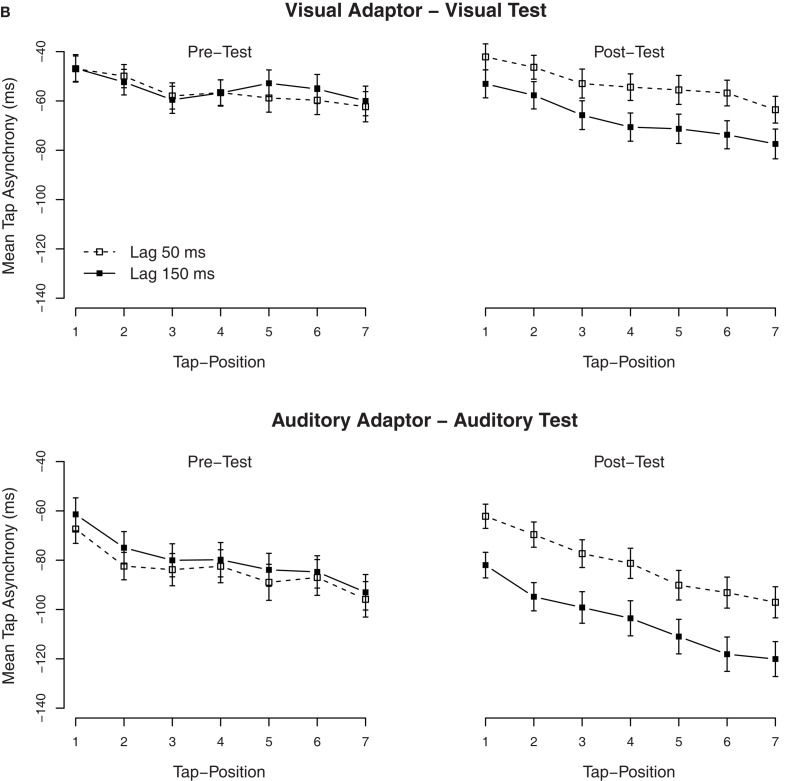
**Mean tap asynchronies in Experiment 1**. **(A)** Mean tap asynchronies per trial block. One block contained three consecutive trials. **(B)** Mean tap asynchronies per tap in one trial. One trial contained seven taps.

Secondary analyses were performed to examine the build-up and the dissipation of the TRE. To examine the build-up, we divided the 30 trials of each condition into 10 blocks of three trials each. The mean asynchronies per block are shown in Figure 2A. The effect of lag-adaptation for visual and auditory adaptors (most easily visible as the difference between lag-150 and lag-50 ms in the post-test) was already at plateau from block 2 onward (trials 4–30), while the effect in the first block (trials 1–3) was close to zero. It is also of interest to note that the mean asynchronies in the pre-tests were alike, thus indicating that the TREs were measured from similar baselines. An ANOVA with block order as additional factor confirmed that in the post-test, there was an interaction between the exposure delay × block order, *F*(9, 486) = 2.5, *p* < 0.01. Separate ANOVAs on the tap asynchronies for each trial block with the modality of the test as between-subjects factor and with the exposure delay as within-subjects factor showed that the main effect of the exposure delay was significant under all trial blocks (*p* < 0.001), except for the first block (*p* = 0.19), thus indicating that the effect had quickly built up within the second block of trials (within 60 taps, ~60 s).

To examine the (possible) dissipation of the effect, mean tapping asynchronies were also calculated for each of the seven individual taps in a trial. The mean asynchronies per tap are shown in Figure 2B. As is clearly visible, tap asynchronies became more negative with each tap, but the TRE remained stable over all taps. An ANOVA on the mean asynchronies in the post-test with tap position as additional factor confirmed that there was a significant effect of tap position (the mean asynchrony increased with tap positions, see the right side of Figure 2B), *F*(6, 324) = 50.6, *p* < 0.001, but tap position did not interact with the exposure delay, *F*(6, 324) = 1.4, *p* = 0.22.

#### Variability

Similar analyses were also done on the variability of the tapping responses (the standard deviation) for each condition. The group-averaged standard deviations are shown in Table 2 (upper panel). A mixed-model ANOVA was conducted on the standard deviation of the tap-stimulus asynchronies with modality of the test as between-subjects factor, and with the test type and the exposure delay as within-subjects factors. The ANOVA showed that there was a significant main effect of the test type, *F*(1, 54) = 14.4, *p* < 0.001, indicating the tap-stimulus asynchrony during the post-test was somewhat more variable than during the pre-test (40.8 vs. 38.7 ms, respectively), but no other effects were significant.

**Table 2:** **Mean standard deviation of tap-stimulus asynchronies**.

Experiment	Modality		Lag (ms)	Mean SD of asynchrony (ms)	
	Adaptor	Test		Pre	Post
Experiment 1	Visual	Visual	50	37.3 (0.9)	38.9 (1.3)
			150	38.9 (1.4)	39.6 (1.1)
	Auditory	Auditory	50	39.6 (1.9)	41.5 (2.2)
			150	39.1 (1.6)	43.3 (1.7)
Experiment 2	Auditory	Visual	50	46.1 (4.2)	45.2 (3.9)
			150	45.0 (3.3)	48.5 (4.2)
	Visual	Auditory	50	33.7 (1.8)	35.1 (2.5)
			150	40.6 (2.1)	44.3 (2.3)

*Standard errors of mean (SEMs) are shown in parentheses*.

### Discussion

In Experiment 1, we demonstrated that an exposure to a fixed delay between a voluntary action (a finger tap) and an external sensory feedback signal (a flash or a tone) of that action increased the natural anticipation tendency in a subsequent sensorimotor synchronization task. Observers exposed to a delayed feedback thus tapped earlier, presumably to compensate the lingering effect of adaptation to delay. This is of importance since it is the first demonstration that a synchronization task can indeed serve as a viable and implicit measure of temporal adaptation. The tapping task also allowed us to examine the build-up and dissipation of temporal recalibration more closely. We found that the effect built up very fast as it came to plateau within ~60 tap-stimulus pairs (~60 s). This coincides with previous reports on fast build-up of temporal recalibration (e.g., Aschersleben and Prinz, 1997; Wozny and Shams, 2011; Yamamoto and Kawabata, 2011). The effect also remained stable within trials over the relatively short period of a taps tested here (seven taps, or ~5 s). It is in line with earlier research which has shown that the temporal recalibration cannot dissipate simply with the passage of time (Machulla et al., 2012). The magnitude of temporal recalibration was somewhat greater for auditory than visual adapters (−24 vs. −12 ms, respectively), which is in line with findings that audition dominates vision in temporal processing (Repp and Penel, 2002, 2004). To further examine sensory-specific aspects of temporal recalibration, we tested whether the effect would transfer across the auditory and the visual modalities in Experiment 2. Equal amounts of transfer would be in line with the idea that a single centralized “clock” is recalibrated, whereas unequal transfer points to modality-specific mechanisms.

## Experiment 2

As in Experiment 1, participants first adapted to delayed auditory or visual feedback of voluntary taps during an exposure phase. In a test phase, they then synchronized their taps to a pacing signal presented in the other modality as the feedback stimulus. If a single mechanism underlies motor-auditory and motor-visual temporal adaptation, one expects the effect to readily transfer in both directions.

### Material and methods

#### Participants

Thirty-four participants from Kyushu Sangyo University and 17 participants from Tilburg University (14 female, mean age 20.2, five left-handed, all using a computer mouse by their right hand) were tested. Twenty-six adapted to delayed visual feedback and then were tested with the auditory synchronization task (13 for each lag condition), the other 25 adapted to delayed auditory feedback and then were tested with the visual synchronization task (13 for lag-50 ms and 12 for lag-150 ms condition). All had normal hearing and normal or corrected-to-normal vision. Informed consent was obtained from each participant. The experiment was approved by the Local Ethics Committee of Kyushu Sangyo University and Tilburg University, and followed the declaration of Helsinki.

#### Stimuli and design

Stimuli and design were as in Experiment 1, except that the modality of the adaptor and the test were switched in a trial. There were also fewer trials (20 trials) in each condition since the effect was at that time already at plateau in Experiment 1. The two exposure delays were run with different participants to avoid carryover effects between adaptations to different lags. The whole experiment lasted ~30 min.

### Results and discussion

#### Mean tap-stimulus asynchronies

Trials of the practice sessions were excluded from further analysis. Performance on the catch trials in the lag-adaptation was almost flawless (99.4% correct on average) indicating that participants were indeed looking at the light or listening to the sound during the adaptation phase. Missing responses were only 0.4% in total. Tap asynchronies out of the range from −250 to +100 ms were eliminated from the analysis (1.4% in total).

The group-averaged tap asynchronies are presented in Table 1 (lower panel). As in Experiment 1, all asynchronies were negative and this anticipation tendency was greater with auditory than with visual test signals. Most importantly, the negative asynchrony became more negative after exposure to the delayed visual adaptor combined with the auditory test, but not after exposure to the delayed auditory adaptor combined with the visual test. To test this, tap-stimulus asynchronies were entered into a mixed-model ANOVA with the modality of the test (visual vs. auditory) and the exposure delay (50 vs. 150 ms) as between-subjects factors, and with test type (pre- vs. post-test) as within-subjects factor. There was a main effect of the modality of the test, *F*(1, 47) = 11.4, *p* < 0.01, since the anticipation tendency was greater with the auditory than with the visual test (−79.2 vs. −51.6 ms, respectively). There were also main effects of the test type, *F*(1, 47) = 25.4, *p* < 0.001, an interaction between the test type × the modality of the test, *F*(1, 47) = 6.2, *p* < 0.05, and the test type × the modality of the test × the exposure delay, *F*(1, 47) = 6.4, *p* < 0.05. No other effects were significant (*p* > 0.05).

To analyze the interactions, a TRE was calculated as before and then tested with separate *t*-tests after Bonferroni correction (alpha was set to 0.0125). This showed that there was a significant TRE with the auditory test (after exposure to the visual adaptor; −31 ms), *t*(12) = 6.4, *p* < 0.001, but *no* TRE with the visual test (after exposure to the auditory adaptor; −4.5 ms), *t*(11) = 0.8, *p* = 0.21. There was no significant difference between the pre- and the post-test with the visual test (with the non-delayed auditory adaptor) and the auditory test (with the non-delayed visual adaptor) [*t*(12) = 1.4, *p* = 0.10, and *t*(12) = 1.9, *p* = 0.04, respectively]. To test directly if the tap asynchrony under the delayed adaptor became more negative compared to the non-delayed adaptor, we ran another *t*-tests (one-sided) on the tap asynchronies between the delayed and the non-delayed adaptor for the visual and the auditory modalities (with Bonferroni corrected alpha set to 0.025). The tap asynchrony with the auditory test after the delayed visual adaptor was more negative than that after the non-delayed one, *t*(24) = 3.4, *p* < 0.01, but the tap asynchrony with the visual test after the delayed auditory adaptor was not significantly different from that after the non-delayed one, *t*(23) = −0.5, *p* = 0.69.

#### Build-up and dissipation

**Figure 3:**
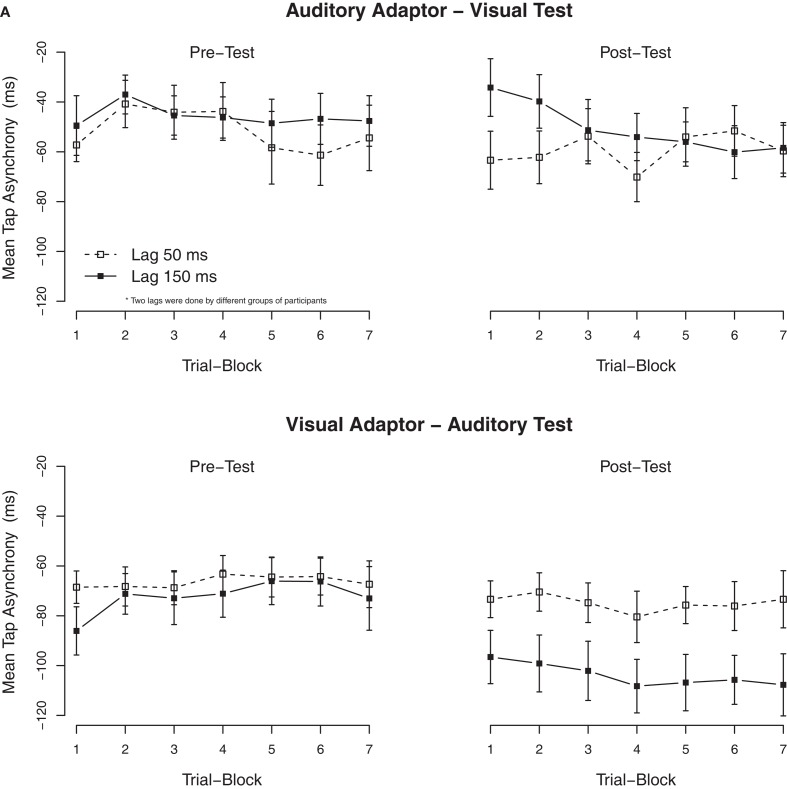

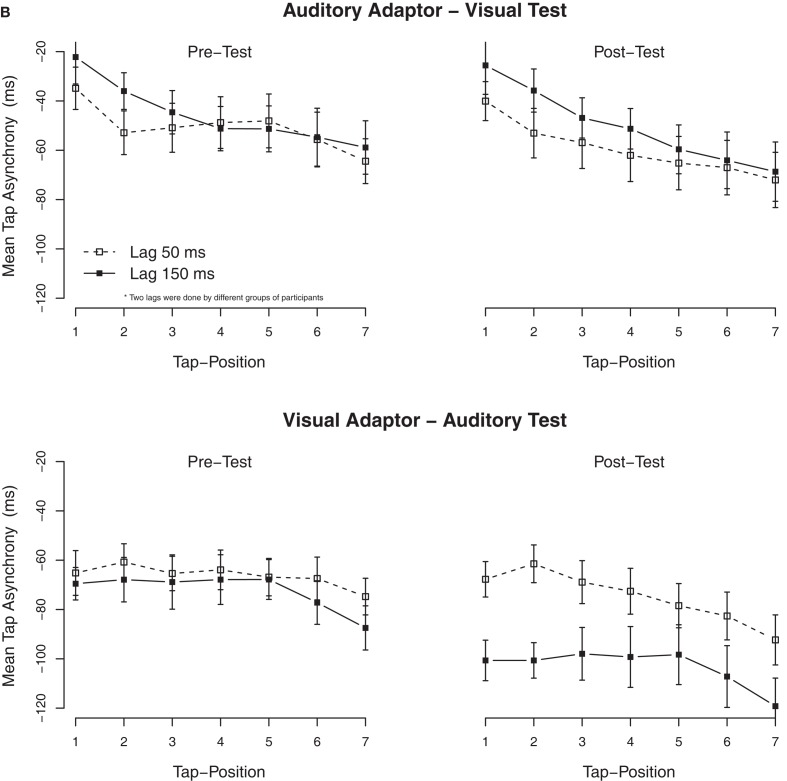
**Mean tap asynchronies in Experiment 2**. **(A)** Mean tap asynchronies per trial block. One block contained three consecutive trials (the last block contained only two trials). **(B)** Mean tap asynchronies per tap in one trial. One trial contained seven taps.

As Experiment 1, secondary analyses were performed to examine the build-up and the dissipation of the adaptation to delays. We divided the 20 trials of each condition into seven blocks of three trials each, except for the last block that only contained two trials. The mean asynchronies per block are shown in Figure 3A. ANOVAs on the tap asynchronies in the post-test, separated by the modality of the test, with the exposure delay and the block order as factors showed a significant effect of the exposure delay in the auditory test (adapted to the visual delay), *F*(1, 24) = 5.2, *p* < 0.05, but not in the visual test (adapted to the auditory delay), *F*(1, 23) = 0.5, *p* = 0.51. Although, an interaction between the exposure delay × the block order was significant in the visual test, *F*(6, 138) = 2.7, *p* < 0.05, subsequent ANOVAs separated by blocks revealed that the effect of the exposure delay was not significant in all trial blocks (*p* > 0.05).

To examine the (possible) dissipation of the effect, mean tapping asynchronies were re-calculated for each of the seven taps in a trial. The mean asynchronies per tap are shown in Figure 3B. As in Experiment 1, the TRE with the auditory test (adapted to the visual delay) remained stable over all taps. An ANOVA on the mean asynchronies in the post-test with tap position as additional factor confirmed that there was a significant effect of tap position, *F*(6, 282) = 17.1, *p* < 0.001, but tap position did not interact with the exposure delay, *F*(6, 282) = 0.1, *p* = 0.99.

#### Variability

The group-averaged standard deviations of the tap asynchronies are shown in Table 2 (lower panel). They were entered into a mixed-model ANOVA with the modality of the test and the exposure delay as between-subjects factors, and the test type as within-subjects factor. There was a significant main effect of the modality of the test, *F*(1, 47) = 6.6, *p* < 0.05, since asynchronies were more variable for visual than auditory test (SDs were 46.2 vs. 38.5 ms, respectively), reflecting a smaller temporal accuracy in the sensorimotor synchronization with visual stimuli as compared to synchronization with auditory stimuli which has been found in earlier studies (e.g., Kolers and Brewster, 1985; Repp and Penel, 2002, 2004; Repp, 2005). No other effects were significant (*p* > 0.05).

To summarize, the main finding was that an exposure to delayed visual feedback increased the anticipation tendency on an auditory synchronization task, but delayed auditory feedback had no effect on a visual synchronization task. This asymmetric transfer points to modality-specific aspects of temporal recalibration.

## General Discussion

In the current study, we sought to examine two questions. Firstly, we examined whether a sensorimotor synchronization task (i.e., tapping in synchrony with a pacing signal) could serve as a viable measure of temporal recalibration. The results clearly indicate that this is indeed the case. We found that taps to an external pacing signal came earlier after being exposed to delayed auditory or visual feedback compared to pre-exposure. No such pre-post exposure difference was found for non-delayed exposure. Presumably this occurred since participants, once adapted to delayed feedback, compensated for this delay by tapping earlier. This speaks to the perceptual nature of temporal recalibration, given that tapping is an implicit measure of synchronization that is free of explicit response biases.

Secondly, we examined whether temporal recalibration would transfer if the modality of the feedback signal and the pacing signal were switched. Here, we found asymmetric effects: exposure to delayed visual feedback affected auditory tapping, but not the reverse. This result appears to contradict earlier studies in which transfer across the auditory and visual modalities has been obtained (Heron et al., 2009; Sugano et al., 2010). These authors used TOJ and/or SJ tasks and reported that temporal recalibration did transfer across motor-visual and the motor-auditory domain in both directions. Sugano et al. (2010) interpreted this finding as a shift of the perceived timing of the motor component: participants may have adjusted their timing criterion of the motor response (when did I move my finger or touch the pad?). Clearly, the present data cannot be explained by a shift in the motor component; if the perceived timing of the motor action was changed, one would expect either a uniform transfer of adaptation across the auditory and visual test stimuli, or possibly even a bigger effect after exposure to auditory rather than visual delayed feedback, since audition is more dominant in time (and might have “attracted” the motor component more). Note, though, that the latter effect was *not* observed here, as we only obtained transfer with the visual adaptor, but not with the auditory adapter.

Our findings therefore suggest that there are also modality-specific aspects at play during adaptation to delayed auditory and visual feedback. One possibility, so far untested, is that during motor-visual adaptation, the *motor* response shifted causing transfer to the motor-auditory domain, while during motor-auditory adaptation it was *audition* that shifted (thus without consequences for the motor-visual domain). Although this explanation is in contrast with the idea that audition is the dominant (and most rigid) modality in time perception, there is some evidence showing a comparable asymmetry. Navarra et al. (2009) showed that participant’s RTs to unimodal auditory stimuli were sped-up after exposure to VA pairs (auditory-late) and RTs were delayed after exposure to AV pairs (auditory-early). Crucially, no such changes were shown in RTs to visual stimuli. The authors explanation was that visual information gives a more exact estimate of the time of occurrence of distal events than auditory information (due to the fact that the time of arrival of visual information regarding an external event is always closer to the time at which this event occurred), and so the visual system might code temporal information more precisely and becomes the dominant modality (Navarra et al., 2009). The data in the present study seem in line with this explanation. In the case of a motor-visual adaptor pair, the visual component is dominant and forces the motor component to shift (thus causing transfer to motor-auditory test pairs), while in case of a motor-auditory adaptor pair, the auditory component shifts (causing no transfer in the motor-visual test pair). Though, to further disentangle which component shifts, the motor or sensory, it would be helpful in future studies if shifts in the involved modalities could be isolated (i.e., for example by testing whether the recalibration effect carries over to tapping by the fellow hand).

Another potential explanation for the asymmetric transfer of TREs may be found in a study by Meyer et al. (2007) who showed that salient sensory stimuli of one modality are “echoed” into other modalities even when apparently unrelated. More specifically, an effect in auditory association cortex was found when stimulation was purely visual (i.e., two red flashes). From this perspective, visual stimuli can evoke activation in auditory association cortex, while auditory stimuli may not evoke a similar response in visual cortex (but, see McIntosh et al., 1998). Moreover, it has been shown that visually presented rhythmic signals are encoded by an auditory code in the brain (Collier and Logan, 2000; Guttman et al., 2005; but, see Grahn et al., 2011). Such an asymmetry in cross-sensory representations might help to explain our asymmetry in the crossmodal transfer of temporal recalibration: the rhythmic visual component of a motor-visual adaptor pair might echo into the auditory modality and induce a motor-“auditory” TRE which then affects the motor-auditory test. On the other hand, a motor-auditory adaptor pair might not (or less strongly) evoke an echo in the visual modality, resulting in no transfer of the TRE to motor-visual test pairs.

Besides explaining the asymmetry of the TREs itself, the present data also raise the question why a motor-auditory adaptor stimulus does not have an effect on a motor-visual tapping task, while it does induce a TRE when a TOJ task is performed (Sugano et al., 2010). One plausible explanation lays in the nature of the two tasks. In the study of Sugano et al. participants perform a TOJ task; a task that automatically forces the participant’s attentional focus toward the temporal structure of a stimulus. In a tapping task on the other hand, probably not the temporal structure but rather the motor-part of the stimulus is the focus of the participant’s selective attention. In a recent study, Heron et al. (2010) have shown that selective attention toward the temporal structure of an adaptor stimulus magnifies TREs in a TOJ task. Whenever participants were instructed to specifically attend to non-temporal features of the adapting stimuli, TREs were substantially smaller. Based on Heron’s finding, we suggest that the diversion in results might be attributed to a difference in attentional states that participants have while performing the different tasks (i.e., TOJ or tapping). As a potential underlying mechanism, Heron et al. proposed that attending to the temporal structure of an adaptor pair might induce a perceptual expansion of the temporal interval between the adaptor components, which then in turn enhances the temporal recalibration process. Following this rationale for the data of the present study, no such expansion of the temporal interval is in play when performing a tapping task, which might have resulted in the null effect when adapting to the motor-auditory pair (apparently, the temporal interval between the motor and visual stimulus is strong enough to induce a measurable TRE on a motor-auditory tapping task, even without the perceptual “expansion”).

When making predictions about the motor or sensory nature of the TRE, it is important to consider that the motor component (i.e., finger tap in the present study) might be decomposed into an intention to make a movement, followed by the actual motor command and an efferent copy of that command. Then, while the finger is moving, the perceiver also receives proprioceptive feedback about the finger movement and the position of the joints, and tactile feedback at the moment that the finger touches an object. So when discussing the possibility of a shift in the motor component, one should be cautious and take into account which part of the motor component shifts. For example, several studies have shown that the perceived timing of the intention of actions is quite flexible (Lau et al., 2007; Nijhawan, 2008; Haggard and Tsakiris, 2009) while it has also been demonstrated that the timing of touch itself is quite rigid (Miyazaki et al., 2006; Harrar and Harris, 2008; Harris et al., 2009). According to these findings, a possible candidate for the shift might be the perceived timing of the motor command rather than the timing of touch. Concerning the somato-sensory feedback part of a motor action, Stenneken et al. (2006) have shown that de-afferented participants (i.e., without somato-sensory feedback) show no asynchrony between finger taps and auditory pacing signals (with visual monitoring), while control subjects show a typical negative asynchrony between taps and pacing signals. These findings demonstrate that somato-sensory information plays a crucial role in the anticipatory timing of a tapping movement. Though, since the somato-sensory part of a motor action is hard to avoid (or delay) in the tapping task that was used in the present study, it is at this time impossible to draw any conclusions about how this somato-sensory feedback is exactly involved in temporal recalibration. In order to disentangle the contribution of each the different motor-components to TREs, future studies might find answers when for example comparing TREs in active-vs.-passive motor tasks, with-or-without touching a surface, or switching hands between adaptor and test stimulus.

It is also of interest to note that the variability of the tap-stimulus asynchronies were not different for the different exposure lags, indicating that the tapping was equally difficult for the delayed feedback condition (lag-150 ms) and the control condition (lag-50 ms). Some researchers have suggested that a lowered sensitivity to lags is the first stage of the temporal recalibration (Navarra et al., 2005; Winter et al., 2008). However, the present results do not support this hypothesis, and rather suggest that temporal recalibration occurs without changing the stability of tapping. This is in line with the earlier studies using TOJ task which have shown that changes in the PSS can occur without changes in the JND after lag-adaptation (Hanson et al., 2008; Sugano et al., 2010; Stekelenburg et al., 2011).

## Concluding Remarks

In this study we demonstrated that a sensorimotor synchronization task (tapping) can be used as a viable measure of temporal recalibration. Observers who have adapted to delayed feedback of a voluntary action (a finger tap) compensated for this delay in a subsequent tapping task by tapping earlier to a pacing signal. This implies that tapping can be added to the available repertoire of tests (besides TOJ and SJ tasks) that measure adaptation to intersensory delays. The most important advantage of tapping is that it is, in contrast with TOJ and SJ tasks, a very easy, fast, and implicit task that even children could do. These advantages should allow researchers to address many new questions.

## Conflict of Interest Statement

The authors declare that the research was conducted in the absence of any commercial or financial relationships that could be construed as a potential conflict of interest.
